# Prominent Grazing Rates and Feeding Preferences of an Abundant Exotic Benthic Herbivore in the Mediterranean Sea

**DOI:** 10.1002/ece3.71686

**Published:** 2025-07-24

**Authors:** Maria Pizarro‐Borrull, Elisabet Font, Núria Marbà, Andrea Anton

**Affiliations:** ^1^ Global Change Research Group, IMEDEA (CSIC‐UIB) Mediterranean Institute for Advanced Studies Esporles Illes Balears Spain; ^2^ International University Menéndez Pelayo Madrid Spain; ^3^ Faculty of Bioscience Autonomous University of Barcelona Barcelona Spain

**Keywords:** decapod, grazing, herbivory, invasive species, non‐native species, *Percnon gibbesi*

## Abstract

Exotic herbivores can exert profound impacts on terrestrial communities, but their ecological effects on marine habitats are not sufficiently quantified. The exotic crab *Percnon gibbesi* , which is rapidly spreading throughout the Mediterranean Sea, grazes almost exclusively on benthic macrophytes, providing an opportunity to study the potential impacts of herbivores in the marine realm. Here, we first quantified the abundance of *P. gibbesi* in Mallorca (Balearic Islands; Spain) in 2023/2024 and reported average densities of 61 individuals 100 m^−2^, approximately 30 times greater than those recorded in 2003 on the islands. We then performed a feeding preference experiment using common native and invasive species of macroalgae (*Caulerpa cylindracea*, *Halimeda incrassata* , *Haliptilon virgatum*, *Halopteris scoparia* , *Padina pavonica*, and *Ulva compressa* ) from the Mediterranean Sea. The per capita grazing rates of *P. gibbesi* (3.83 ± 1.71 WW g crab^−1^ day^−1^), which can ingest almost 75% of their body weight daily, were higher than those recorded for most native herbivorous species in the Mediterranean. The estimated daily grazing rates for *P. gibbesi* average 23.59 ± 15.17 kg WW macroalgae ha^−1^ day^−1^, value that corresponds with 0.1% to 10.9% (average 5.5%) of the total macroalgae production in this area. Our experiment revealed clear preferences of *P. gibbesi* for three species of macroalgae, which were not explained by the nutritional content. Overall, our results generate great concern and, coupled with the large extent of the invasion, indicate that the ecological impacts of this exotic herbivore on Mediterranean marine communities could be substantial, and have, until now, gone largely unreported.

## Graphical abstract

In this study, we report the potential negative impacts of an exotic herbivore crab ( *Percnon gibbesi* ) in Mediterranean benthic habitats. Given the high densities of *P. gibbesi* found in the field, the large per capita and per area grazing rates quantified experimentally, and the broad generalist diet reported here and elsewhere, the ecological impacts of *P. gibbesi* on shallow rocky shore ecosystems of the Mediterranean Sea are expected to be severe.

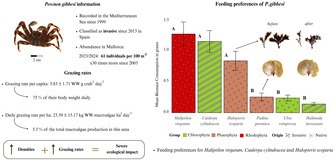

## 1 Introduction

Invasions by herbivores represent a serious threat to native communities (Nuñez et al. 2010). For example, the introduction of the beaver *Castor canadensis* in Tierra del Fuego (Chile and Argentina) produced long‐term changes in the composition and succession of herbaceous communities (Wallem et al. 2010). Similarly, the introduction of the back‐tailed deer *Odocoileus hemionus* to islands of British Columbia exerted a strong top‐down effect, where not only plants but also invertebrate and shrub‐dependent songbird communities were greatly simplified (Martin et al. 2010). In marine systems, which tend to be strongly regulated by top‐down forces (Shurin et al. 2006), species interactions such as herbivory can be particularly important: a global study showed that marine herbivores can strongly reduce producer abundance by an average of 68% (Poore et al. 2012). The introduction of herbivorous species, such as rabbitfish species ( *Siganus rivulatus* and *S. luridus* ) in the western Mediterranean, can drive shifts in grazing patterns that can increase the vulnerability of native macrophytes (Santana‐Garcon et al. 2023). This direct impact of invasive herbivores on primary producers can also drive indirect cascading effects on the biodiversity and ecosystem services provided by native communities. For example, the invasion of the sea urchin *Centrostephanus rodgersii* in eastern Tasmania decreased macroalgae abundance creating barrens that negatively affected the populations of other commercially important native herbivores (e.g., abalone *Haliotis rubra* and urchin *Heliocidaris erythrogramma*; Strain and Johnson 2009). Despite the potentially devastating ecological impacts of marine invasive herbivores, a recent meta‐analysis shows that the effects of only a few species of grazers have been quantified worldwide (Anton et al. 2019, 2020).

The Mediterranean Sea, a hot spot of marine biodiversity with almost 17,000 native marine species (Coll et al. 2010), is the most invaded marine region of the world, with approximately 1000 exotic marine species, including several species of herbivores (Katsanevakis et al. 2011; Tsirintanis et al. 2022; Zenetos et al. 2020; Roy et al. 2024). The crab *Percnon gibbesi* , a tropical and subtropical benthic grazer native of both sides of the Atlantic Ocean and the Pacific coast of North America (Manning and Holthuis 1981), is rapidly spreading throughout the Mediterranean Sea (Zenetos et al. 2005; Cannicci et al. 2008; Katsanevakis 2020). It was first reported in 1999 simultaneously in Italy (Relini et al. 2000) and the Balearic Islands (Garcia Ll 2000; Müller 2001). Despite its ubiquitous presence, only a few studies exist on the basic ecology of *P. gibbesi* (Deudero et al. 2005; Puccio et al. 2006; Tiralongo et al. 2021; Bada et al. 2022; Puentes and Anton 2025). *Percnon gibbesi* was measured at 2 individuals per 100 m^−2^ around Mallorca in 2003 (Deudero et al. 2005). In southeast Sicily, *P. gibbesi* was the most abundant prey for rock goby *Gobius paganellus* (Tiralongo et al. 2021). Three studies (Puccio et al. 2006; Bada et al. 2022; Puentes and Anton 2025) document *P. gibbesi* as an almost exclusive herbivore. A recent publication reported that *P. gibbesi* can consume endemic seagrass *Posidonia oceanica* , including its leaves, fruits, and rhizome (Puentes and Anton 2025). Puccio et al. (2006) reported that the diet of *P. gibbesi* in Sicily was primarily filamentous, calcareous, and corticated macroalgae. In Algerian waters, Bada et al. (2022) determined that 95.2% of the stomach contents of *P. gibbesi* were benthic macrophytes, principally coralline red algae (e.g., *Ellisolandia elongate* and *Jania longifurca* ), and filamentous brown alga (e.g., *Halopteris scoparia* ). Although *P. gibbesi* is widely distributed in the Mediterranean Sea, no studies have quantified its potential ecological impact on macroalgae, and its natural density has been barely quantified (Deudero et al. 2005).

The overall aim of this study was to assess the potential ecological impact of an exotic herbivore on marine benthic habitats. The specific objectives of our field and laboratory studies were to measure the abundance and feeding ecology of the exotic herbivorous crab *P. gibbesi* . We first quantified the abundance of *P. gibbesi* in two locations in Mallorca during the summer (July/August on 2023 and 2024), when *P. gibbesi* appears to feed more actively (Authors, personal observation). We also performed a laboratory experiment to quantify the per capita grazing rates and feeding preferences of *P. gibbesi* with six common native and exotic macroalgal species (*Caulerpa cylindracea*, *Halimeda incrassata* , *Haliptilon virgatum*, *Halopteris scoparia* , *Padina pavonica*, and *Ulva compressa* ). We also estimated the potential consumption rates of *P. gibbesi* per hectare by combining their mean per capita feeding rates, obtained experimentally in the laboratory, with the abundance of *P. gibbesi* quantified in field surveys conducted in the summer in Mallorca.

## 2 Materials and Methods

### 2.1 Field Summer Surveys of *Percnon gibbesi*

**FIGURE 1:**
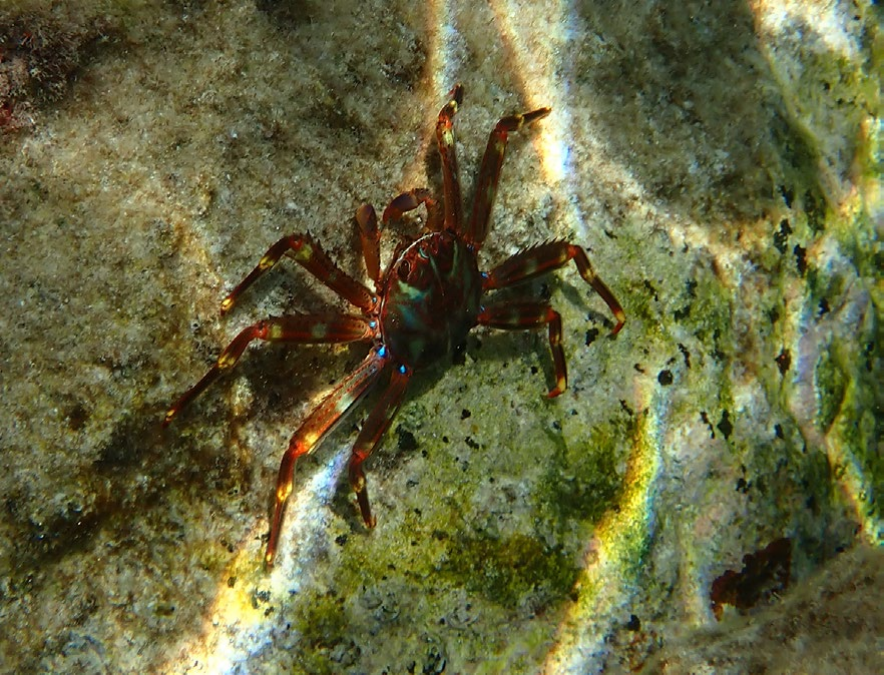
Picture of *Percnon gibbesi* photographed in a rock in Cala Estellencs (Mallorca; Balearic Islands; Spain). Picture credit: Maria Pizarro‐Borrull.

We quantified the abundance of *P. gibbesi* in two locations along the north‐west coast of Mallorca, Spain (Port of Valldemossa N 39.71752, E 2.58721 and Cala Estellencs N 39.65888, E 2.47149) in July/August of 2023 and 2024 (Figure 1). The number of *P. gibbesi* was counted along a transect parallel to the shore following (Deudero et al. 2005). In each location, three to eight transects (30 m long and of 2 m wide) were deployed on hard substrates‐mainly boulders and rocks often covered with algae‐ and *P. gibbesi* densities were quantified visually at depths of approximately 0.5–3 m.

### 2.2 Per Capita Grazing Rates and Feeding Preferences in a Laboratory Experiment

The multichoice feeding preference experiment was performed using six species of macroalgae. Three of them are common native species during the summer in the Balearic Islands: the red calcareous algae *Haliptilon virgatum*, the brown fleshy alga *Halopteris scoparia* , and the brown calcareous alga *Padina pavonica* (representing approximately 25%, 27%, and 25% of the macroalgae biomass respectively in July/August 2023 in Cala Estellencs; Author, unpublished results). The fourth native species used was the green fleshy alga *Ulva compressa* , which was not present in Cala Estellencs in the summer of 2023 but was abundant in shallow rocky areas of the Port of Valldemossa when we performed the *P. gibbesi* survey counts in July 2023. Finally, we included in the experiment two exotic macroalgal species, the green fleshy *Caulerpa cylindracea* (widely distributed in the Mediterranean; Anton et al. 2019) and the green calcareous *Halimeda incrassata* (widely distributed in Mallorca and Cabrera; Mateo‐Ramírez et al. 2022; Authors, personal observation).

Individuals of *P. gibbesi* were collected by hand on the 10^th^ of July 2023 with the help of fishing nets from rocky crops in Cala Estellencs, Mallorca (N 39.65888, E 2.47149) and transported in coolers to IMEDEA within 2 h of collection. The macroalgae *Haliptilon virgatum*, *Halopteris scoparia* , *Padina pavonica* and *Ulva compressa* were also collected by hand. The exotic macroalgae *Caulerpa cylindracea* and *Halimeda incrassata* were collected by hand from the nearby island of Cabrera (N 39.15295, E 2.94630). The crabs were allowed to acclimate individually for 5 days in aeriated 15 L aquaria equipped with a water filtration system at temperatures ranging from 25°C to 26°C and a salinity of 37.5, which were the temperature and salinity values at the time of collection. Crabs were fed *Halopteris scoparia* ad libitum every day during the acclimation period. Half of the water in the aquaria was replaced every 2 days after the bottom was cleaned with the help of a syphon. Macroalgae were kept in five additional 15 L aquaria under similar temperature and salinity conditions as those in the tanks with crabs.

Before the start of the feeding preference experiment, the temperature of the aquaria was raised to 26.5°C ± 0.4°C within 24 h. This experimental target temperature was the mean summer temperature measured in situ every 10 min in Cala Estellencs from June to August 2023 with a miniDOT permanently deployed at 0.5–1 m water depth (e.g., 26.57°C ± 2.21°C; mean ± SD). Crabs were subjected to a light cycle of 14:10 as in summer conditions in the Balearic Islands and were exposed to 150 μmol m^−2^ s^−1^ photosynthetically active radiation (PAR) during the daylight hours. Salinity was checked and adjusted with Milli‐Q water every other day, and the concentration was maintained at 37.5 ± 0.3 ppm. Two days before the beginning of the experiment, the crabs were not fed to standardize hunger levels across individuals as previously done with crabs (Fieber and Bourdeau 2021; Bergamino and Richoux 2015) and other marine invertebrates (Pagès et al. 2018). For the experiment, 12 crabs (six males and six females) were utilized. Their wet weight and carapace width were measured before the experiment with the help of a scale and a Vernier caliper.

A 24 h feeding preference experiment was carried out following the design proposed by Prince et al. (2004), where the control and experimental treatments were nested within the same aquarium. Two hand‐made identical net cages (18 × 9 × 18 cm^3^ and mess size 1 mm^2^) were included in each aquarium, one for the control with the six macroalgae and one for the experimental treatment with the six macroalgae and one crab. The cage location per aquarium (e.g., top or bottom) was randomly allocated. Two grams of each macroalgal species were placed in all the cages. Macroalgae wet weight was assessed before and after the 24 h feeding experiment after five handshakes and spinning 20 times in a salad spinner (Prince et al. 2004). The total and species consumption rates per crab were estimated as the difference in macroalgal weight during the experiment divided by the duration of the experiment (1 day).

### 2.3 Nutritional Quality of the Macroalgae

The nutritional quality of the macroalgae was assessed in the blades of the six species. Specimens of each species of macroalgae were dried in paper envelopes in a stove at 60°C and then ground into powder using an agate mortar and pestle. Organic carbon and nitrogen contents (%C_org_ and %N) were analyzed in five replicates per species of macroalgae using Carlo‐Erba elemental analysis. Organic phosphorous content (%P) was quantified in two replicates for each of the six species of macroalgae using inductively coupled plasma mass spectrometry (ICP‐MS). Inorganic carbon content (%C_inorg_) of the species of calcareous macroalgae was assessed in four replicates per species using an acidification process by dissolving the inorganic carbon content from the blades of the macroalgae using 4 M HCl. The percentage of inorganic carbon content was determined by subtracting the initial biomass from the final biomass after the acidification process.

### 2.4 Statistical Analyses

We performed a Wilcoxon test to compare the abundance of *P. gibbesi* between locations and years. In the multiple‐choice feeding experiment, we followed the design proposed by Prince et al. (2004) to overcome previously detected statistical limitations when more than two food types were used, such as the use of inappropriate control treatments (Peterson and Renaud 1989), or the use of a constant correction across replicates to account for autogenic changes (Roa 1992). We paired control and experimental treatments within the same aquarium to account for autogenic changes (e.g., changes in macroalgae biomass unrelated to crab consumption) at the aquarium level while including appropriate unique control treatments. Changes in macroalgae biomass in the control cage were used to correct biomass consumption in experimental treatments (e.g., changes in macroalgae biomass in the treatment cage—changes in macroalgae biomass in the control cage) before performing statistical analysis (following Prince et al. 2004). A multivariate analysis on the basis of the means of consumption was performed using a Hotelling's *T* ^2^ followed by a post hoc pairwise Wilcoxon comparison test (Prince et al. 2004) to assess significant differences between macroalgal species. The effect of macroalgae taxonomic group (e.g., red, green, or brown) on biomass consumption by the crab *P. gibbesi* was determined with a Kruskal–Wallis test, followed by a post hoc pairwise Wilcoxon comparison test. The same type of analysis was performed to test for the effect of the origin of the macroalgae (e.g., native vs. exotic) on biomass consumption by the crab *P. gibbesi* . To explore the potential relationships between the total macroalgae biomass consumption and the biological parameters of the crab (e.g., body weight, carapace length, and sex), two simple linear regressions (for body weight and carapace length), and a one‐way ANOVA (for sex) were performed. To assess if nutrient content of the macroalgae blades (%N, %P, %C_org_, C/N, C/P, and %C_inorg_) could influence macroalgae consumption rates, six simple linear regression analyses were performed. All the statistical analyses were performed in R.

## 3 Results

**FIGURE 2:**
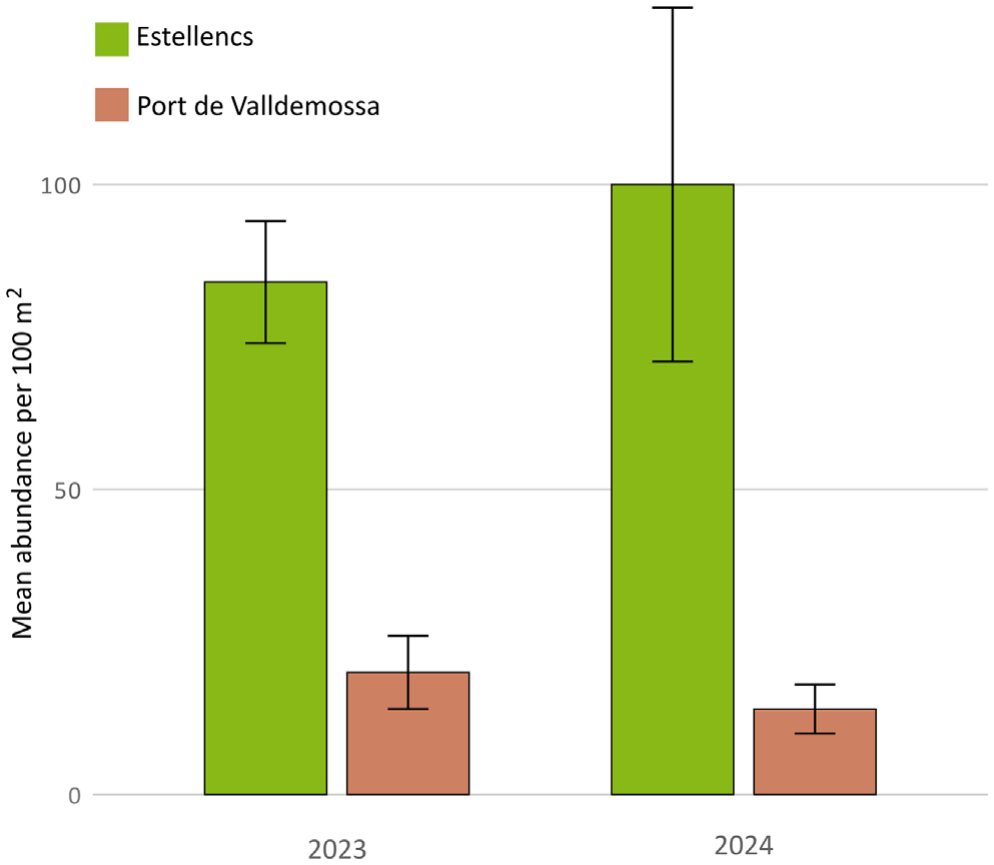
Abundance of *Percnon gibbesi* per 100 m^−2^ over 2 years (2023 and 2024) in Mallorca. Bars represent the mean (± SE; *n* = 3–8 transects per site and sampling time) abundance of *Percnon gibbesi* per 100 m^−2^ in Port de Valldemossa (pink) and Cala Estellencs (green).

The average density of *P. gibbesi* for two locations in north‐western Mallorca (Balearic Islands, Spain) in 2023/2024 was 61.4 ± 53.78 crabs 100 m^−2^ (mean ± SD; Figure 2). During the summer of 2023 at the two shallow rocky sites, the density of crabs was 66.5 ± 38.3 crabs per 100 m^−2^ being on average more than 4‐fold greater in Estellencs (84 ± 28 crabs per 100 m^−2^) than in Port de Valldemossa (20 ± 10 crabs per 100 m^−2^, Figure 2). During the summer of 2024, the average abundance of *P. gibbesi* was 56.7 ± 66.4 crabs 100 m^−2^, similar to that recorded in 2023 (Figure 2). Likewise, the abundance of *P. gibbesi* accounted in Port de Valldemossa (14 ± 10 crabs per 100 m^−2^) in 2024 was smaller than that detected in Estellencs 100 ± 72 crabs 100 m^−2^ (Figure 2). There was not a significant difference in the abundance of *P. gibbesi* among years (2023 vs. 2024; *p*‐value: 0.267, Wilcoxon test), but there was a significant difference among study locations (Estellencs vs. Port de Valldemossa: *p*‐value: 0.0025, Wilcoxon test).

**FIGURE 3:**
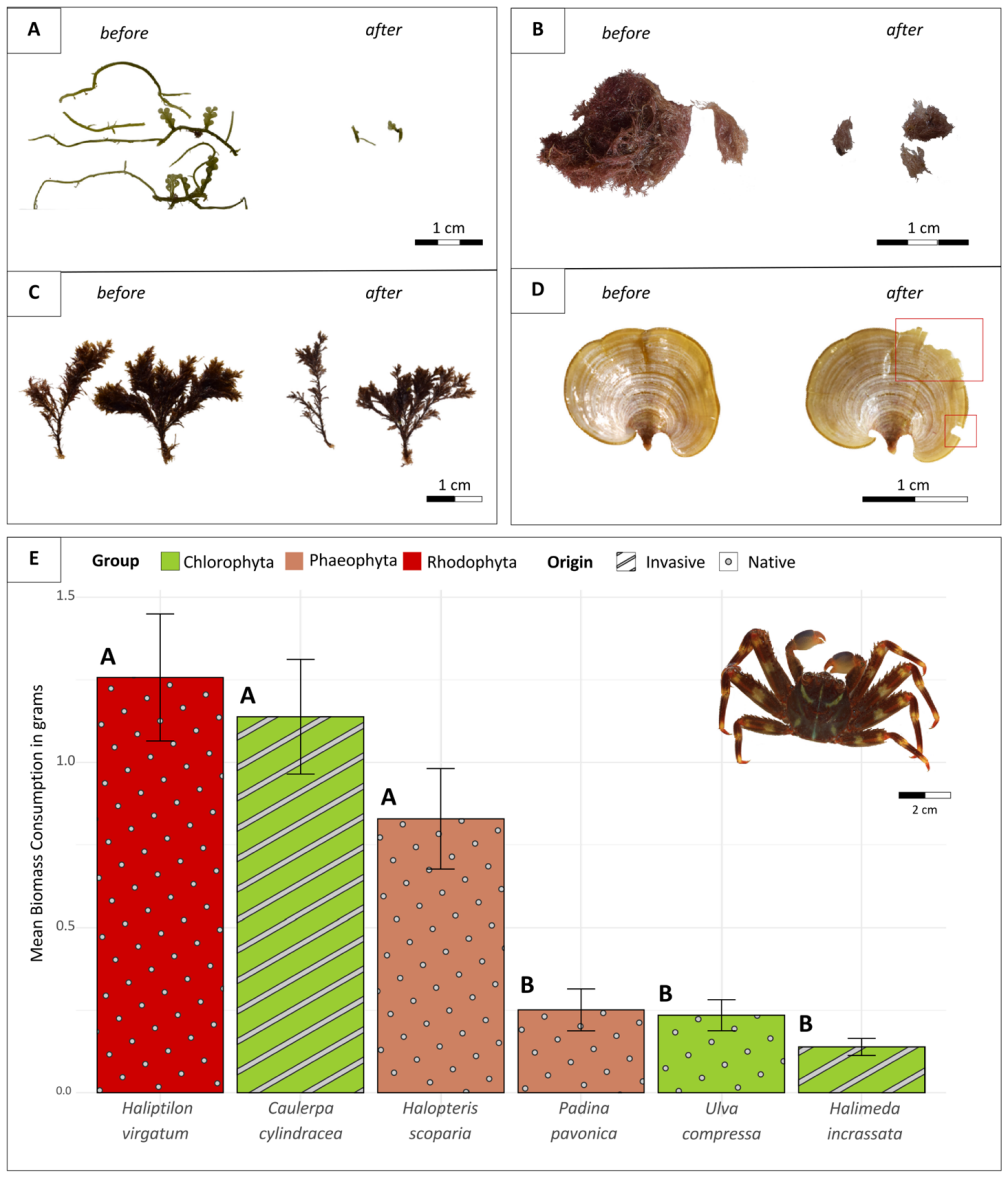
Macroalgae showing signs of herbivory (A) *Haliptilon virgatum*, (B) *Caulerpa cylindracea*, (C) *Halopteris scoparia*, and (D) *Padina pavonica* before and after the 24 h experiment in the presence of *Percnon gibbesi* . Clear feeding marks are highlighted with a red square in *Padina pavonica* (red square in D). (E) Total biomass consumption (mean ± SE) in grams per crab per day for each macroalgal species. Capital letters indicate significant differences at *p*‐values < 0.05 in post hoc pairwise Wilcoxon comparisons.

In the feeding preference experiment, all macroalgae were collected from the treatment cages after 24 h. These macroalgae showed clear signs of herbivory, such as fragmentation (Figures 3A and 2B,C) or bite marks in the blades (Figure 3D), which resulted in a decrease in macroalgae biomass. There was a significant difference in biomass consumption among the six macroalgal species (*p* = 0.001, *T* ^2^ = 18.76, *F* = 18.76, df = 6, Hotelling's *T* ^2^ test), with *P. gibbesi* showing a preference for *H. virgatum* , *C. cylindracea* and *H. scoparia* , whereas the less preferred species were *U. compressa* , *H. incrassata* , and *P. pavonica* (post hoc pairwise Wilcoxon comparisons are indicated in capital letters in Figure 2E and the legend).

There were significant differences between macroalgae taxonomic groups (e.g., red, green, and brown algae) with respect to mean biomass consumption (*p*‐value = 0.0022, *χ* ^2^ = 12.16, df = 2, Kruskal–Wallis test). Post hoc comparisons revealed significant differences in the consumption between Rhodophyta and Chlorophyta (*p*‐value = 0.004, pairwise Wilcoxon test) and between Rhodophyta and Phaeophyta (*p*‐value = 0.0056, pairwise Wilcoxon test) but no significant differences between Phaeophyta and Chlorophyta algae consumption (*p*‐value = 1, pairwise Wilcoxon test). In addition, *P. gibbesi* did not consume more native macroalgae than invasive macroalgae (*p*‐value = 0.5189, *χ* ^2^ = 0.4161, df = 1, Kruskal–Wallis test).

The average total macroalgae biomass consumption rate was 3.83 ± 1.71 (mean ± SD) WW g crab^−1^ day^−1^. The daily grazing rates of macroalgae per hectare for *P. gibbesi* were on average 23.59 ± 23.17 (mean ± SD) kg ha^−1^ day^−1^. Daily grazing rates during 2023 were estimated at 25.47 ± 18.56 (mean ± SD) kg ha^−1^ day^−1^, being 7.66 ± 5.13 (mean ± SD) kg ha^−1^ day^−1^ and 32.17 ± 17.92 (mean ± SD) kg ha^−1^ day^−1^ in Port of Valldemossa and Estellencs, respectively. There was a similar daily grazing rate consumption in 2024 with an average of 21.72 ± 23.17 (mean ± SD) kg ha^−1^ day^−1^, being 5.36 ± 4.51 (mean ± SD) kg ha^−1^ day^−1^ in Port de Valldemossa and 38.30 ± 32.44 (mean ± SD) kg ha^−1^ day^−1^ in Estellencs.

Crabs used in the experiment had a mean body weight of 5.842 ± 2.996 g (mean females = 4.974 ± 2.303 g, mean males = 6.710 ± 3.555 g) and a mean carapace width of 1.667 ± 0.025 cm (mean females = 1.6 ± 0.322 cm, mean males = 1.73 ± 0.327 cm), ranging from 1.2 to 2.2 cm. We found no significant differences between males and females for crab weight (*p*‐value = 0.339, *F*‐value = 1.008, df = 1, 10; one‐way ANOVA) and carapace width (*p*‐value = 0.493, *F*‐value = 0.506, df = 1, 10; one‐way ANOVA). The mean grazing rates (scaled to their body weight) for females and males were 76.3% ± 37.15% and 73.13% ± 27.7%, respectively. There was no significant difference in macroalgae biomass consumption between the sexes of the crabs (*p*‐value = 0.903, *F*‐value = 0.016, df = 1, 10; one‐way ANOVA). No relationship was found between macroalgae biomass consumption and body weight (*p*‐value = 0.068, adjusted *R* ^2^ = 0.223, df = 1, 10; linear regression) or carapace width (*p*‐value = 0.2463, adjusted *R* ^2^ = 0.044, df = 1, 10; linear regression) of the crabs.

**FIGURE 4:**
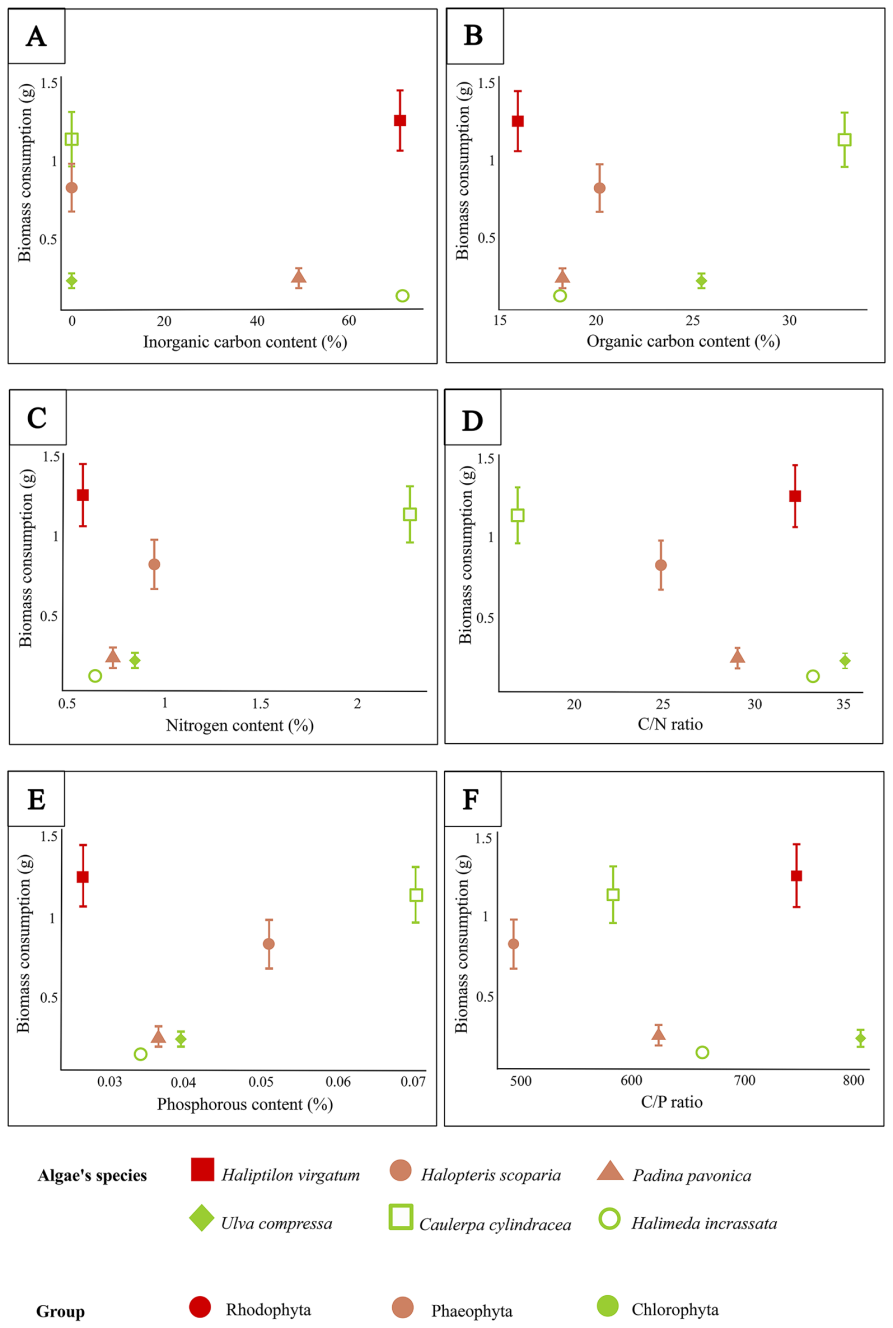
Total mean (± SE) macroalgae biomass consumption for each macroalgal species in relation to the mean (A) inorganic carbon content (%), (B) organic carbon content (%), (C) nitrogen content (%), (D) C/N ratio, (E) phosphorus content (%), and (F) C/P ratio in the macroalgae blades.

There was no relationship between macroalgae inorganic carbon content and the biomass consumed by *P. gibbesi* (*p*‐value = 0.8089, adjusted *R* ^2^ = −0.229, df = 1, 4; linear regression; Figure 4A). Similarly, no relationships were found between macroalgae biomass consumption and organic carbon (*p*‐value: 0.6856, adjusted *R* ^2^: −0.1934, df = 1, 4; linear regression; Figure 4B), organic nitrogen content (*p*‐value: 0.3718, adjusted *R* ^2^: 0.002, df = 1, 4; linear regression; Figure 4C), organic C/N ratio (*p*‐value: 0.2464, adjusted *R* ^2^: 0.1439, df = 1, 4; linear regression; Figure 4D), phosphorous content (*p*‐value: 0.4227, adjusted *R* ^2^: −0.0425, df = 1, 4; linear regression; Figure 4E), or the organic C/P ratio (*p*‐value: 0.677, adjusted *R* ^2^: −0.190, df = 1, 4; linear regression; Figure 4F).

## 4 Discussion

Despite its ubiquitous presence throughout the Mediterranean, there have been relatively few studies on the abundance and feeding ecology of the exotic crab *Percnon gibbesi* (Deudero et al. 2005; Agnetta et al. 2013). The densities of *P. gibbesi* during the summers of 2023 and 2024 in north‐western Mallorca (Balearic Islands, Spain) were significantly higher than the previously recorded densities for this species in the Mediterranean Sea. At two shallow rocky sites, we observed an average density of about 60 individuals of *P. gibbesi* per 100 m^2^ (61.4 ± 53.78 crabs 100 m^−2^) in 2023/2024. This mean density is 31 times greater than that documented in the Balearic Islands in July/August 2003 (2 crabs per 100 m^2^; Deudero et al. 2005), and it is also higher than that recorded in Sicily in 2010 (42 crabs 100 m^−2^; Agnetta et al. 2013), which, to our knowledge, represent the only previous records of *P. gibbesi* density per unit area in the Mediterranean. Compared to other common native benthic herbivores, *P. gibbesi* are 6 to 11 times more abundant than the native sea urchins *Paracentrotus lividus* and *Arbacia lixula* , which have densities of 9.8 and 5.6 urchins per 100 m^2^, respectively, across three sites (Dragonera, Menorca, and Ibiza) in the Balearic Islands in 2024 (Anton 2024).

We present the first experimental quantification of the grazing rates of *P. gibbesi* on macroalgae, which can consume on average per capita 3.83 ± 1.71 WW g crab^−1^ day^−1^ (mean ± 1 SD). To compare this feeding rate with other marine species, we created Table 1, which includes estimates of feeding rates for other marine herbivores and crab species. The per capita feeding rate of *P. gibbesi* is nearly four times higher than the experimentally quantified rates for the purple sea urchin *P. lividus* , a common herbivore in shallow Mediterranean ecosystems. Specifically, the feeding rates for *P. lividus* on two macrophyte species ( *Ulva rigida* and *Posidonia oceanica* ) were 1 ± 0.01 and 0.88 ± 0.18 WW g urchin^−1^ day^−1^, respectively, despite the urchin being nearly five times heavier than the invasive crab. A comparable trend is found in other species of crabs, such as the herbivore *Mithrax spinosissimus* , and the carnivores *Cancer magister* and *Paralithodes camtschatica* , whose consumption rates are 3, 4, and 122 times greater (13.18, 16.8, and 469 WW g crab^−1^ day^−1^; respectively) than those of *P. gibbesi* but are much heavier than those of the invasive crab (500, 150, and 1700 times; respectively; Table 1). Similarly, the fish herbivore *Sarpa salpa* is 125 times heavier than *P. gibbesi* (average body weight 727 g) but consumes only five times more per capita (18.17 g fish^−1^ day^−1^; Table 1). Remarkably, the herbivorous urchin *Strongylocentrotus droebachiensis* consumes (0.18 ± 0.054 WW g urchin^−1^ day^−1^) 21 times less macroalgae than *P. gibbesi* while being four times heavier (average body weight 22.3 g). In all these comparisons, *P. gibbesi* is the smallest species, and its seemingly high consumption rates could be attributed to body‐size allometric scaling (e.g., smaller species have higher metabolic demands). However, the herbivorous amphipod *Gammarellus homari* , 22 times lighter than *P. gibbesi* , consumes 166 times less algae per capita than the invasive crab.

**TABLE 1:** Feeding and consumption rates for worldwide marine herbivores and carnivores compared to invasive *Percnon gibbesi* .

Species	Diet	Feeding rate (g ind^−1^ day^−1^)	Animal weight (g)	Consumption rate (%)	Population density (ind ha^−1^)	Consumption rate per ha (kg ha^−1^ day^−1^)
*Percnon gibbesi* ^a^	H	3.83 ± 1.71	5.84 ± 3.00	75.1 ± 0.31^b^	6140 ± 5378	23.59 ± 15.45^b^ (5.36–38.3)
*Paralithodes camtschatica*	C	469 ± 130 (Zhou et al. 1998)	10,000 (Animal Diversity Web, in press)	4.7	—	—
*Cancer magister*	C	16.8 ± 1.39 (Schultz and Shirley 1997)	874.5 ± 164.09 (Gamblewood et al. 2018)	1.9	—	—
*Mithrax spinosissimus*	H	13.18 (Butler and Mojica 2012)	3000 (Winfree and Weinstein 1989)	0.44	70 (Butler and Mojica 2012)	0.922
*Strongylocentrotus droebachiensis*	H	0.18 ± 0.054 (Wessels et al. 2006)	22.3 ± 4.92 (Wessels et al. 2006)	0.81	140,000 (Scheibling and Hatcher 2013)	25.62
*Paracentrotus lividus*	H	1 ± 0.01 (Ruocco et al. 2018)	27.4 (14.8 (Fernandez and Boudouresque 2000) to 40 (Ruocco et al. 2018))	0.04	20,824 (*n* = 984 (Anton 2024) *n* = 40,720 ± 15,260 (Hereu et al. 2014))	20.82
*Paracentrotus lividus*	H	0.88 ± 0.17 (Ruocco et al. 2018)	27.4 (14.8 (Fernandez and Boudouresque 2000) to 40 (Ruocco et al. 2018))	0.03	20,824 (*n* = 984 (Anton 2024) *n* = 40,720 ± 15,260 (Hereu et al. 2014))	18.32
*Gammarellus homari*	H	0.023 ± 0.01 (Wessels et al. 2006)	0.26 ± 0.09 (Wessels et al. 2006)	8.84		
*Ampithoe longimana*	H	~0.009 (Cruz‐Rivera and Hay 2001)	—	—	5,968,254 (Virnstein et al. 1983)	53.71
*Sarpa salpa*	H	18.17 ± 10.17 (Goldenberg and Erzini 2014)	727 ± 143 (Goldenberg and Erzini 2014)	2.5%	2000 ± 600 (Gianni et al. 2017)	36.2
*Diplodus holbrooki*	O	3.01 ± 0.39 (Hay et al. 1987)	~331.33 (Manooch 1996)	0.9%	75,000 (Hay 1986)	225.75

*Note:* Diet: H: Herbivore; C: Carnivore; O: Omnivore. Feeding rates are expressed as mean ( ± SD) in grams per day, animal weight in grams, consumption rates are scaled to % body weight, population density in number of individuals per hectare, and consumption rate per hectare is expressed as kilogram of food ingested per hectare.

^a^ Our study.

^b^ Calculations made using raw data (12 replicates).

Therefore, when combining the per capita grazing rates with average body weight, we find that *P. gibbesi* can ingest 75% of its body weight daily, a percentage that significantly exceeds those reported for other marine herbivores (e.g., fish *S. salpa* 2.5%, fish *Diplodus holbrooki* 0.9%, crab *M. spinosissimus* 0.44%, urchins *P. lividus* 0.07%, *S. droebachiensis* 0.81%, and amphipod *G. homari* 8.84%; Table 1) or marine carnivorous crabs (e.g., *C. magister* 1.9% and *P. camtschatica* 4.7%; Table 1). Therefore, the consumption rate of exotic *P. gibbesi* relative to its size is at least eight times higher than any other marine species in this comparison, including those smaller than the invasive crab. In fact, this rate exceeds those reported for terrestrial animals with traditionally high metabolic demands, such as shrews and hummingbirds, which can consume up to 50% of their body weight daily (Tilford 2015; Geyer et al. 2022). Our estimates of *P. gibbesi* grazing rates may have been marginally overestimated for two reasons. First, small algal fragments may have been produced during feeding and not ingested by the crabs. Both the crabs and the algae were enclosed in mesh cages during the experiment, and all macroalgal fragments found within the cages were carefully collected using tweezers and included in the final biomass calculations. However, it is possible that very small particles may have passed through the mesh, potentially leading to a slight overestimation of daily feeding rates. Second, the crabs underwent a 2‐day fasting period prior to the feeding preference experiment (a common practice used in similar studies; Fieber and Bourdeau 2021; Bergamino and Richoux 2015; Pagès et al. 2018), which may have increased their feeding activity and thus influenced the recorded grazing rates.

Our calculations of daily grazing rates of macroalgae per hectare for *P. gibbesi* average 23.59 ± 15.45 WW kg macroalgae ha^−1^ day^−1^ (Table 1). These values surpass those reported for many other marine herbivores, such as the spider crab *Mithrax spinosissimus* (0.922 kg ha^−1^ day^−1^; Table 1) and purple sea urchin *P. lividus* (18.32–20.82 WW kg ha^−1^ day^−1^; Table 1) and are similar to those of the green sea urchin *S. droebachiensis* (25.62 WW kg ha^−1^ day^−1^; Table 1). The grazing rates reported for the fish *Sarpa salpa* in the French Mediterranean (36.2 WW kg ha^−1^ day^−1^; Table 1), one of the most prominent and abundant herbivores in the basin (Prado et al. 2007), are also comparable to those estimated for *P. gibbesi* in Estellencs in 2023/24 in this study (35.1 WW kg ha^−1^ day^−1^). The estimated grazing rates of both the amphipod *Ampithoe longimana* (53.71 WW kg ha^−1^ day^−1^), a major herbivore in the USA (Duffy and Hay 2000), and the spottail pinfish *Diplodus holbrooki* in the Western Atlantic coast (226 WW kg ha^−1^ day^−1^; Table 1) exceed those estimated here for *P. gibbesi* . Taking into consideration that average primary production of benthic macroalgae in the intertidal and subtidal in the Mediterranean is 430 ± 409 WW kg macroalgae ha^−1^ day^−1^ (Duarte et al. 2022), *P. gibbesi* could be potentially consuming on average 5.5% (ranging from 0.1% to 10.9%) of the total macroalgae production in this area. Nevertheless, these estimations of consumption rates—per capita or per area—of *P. gibbesi* are based on short‐term experimental results, and further longer‐term and/or in situ investigations are warranted to fully understand the ecological impact of this invader on macrophytes in the Mediterranean.

None of the macroalgal species offered were ignored by *P. gibbesi* , which consumed on average at least 7% of the biomass of any of them in 1 day (Figure 3). These results indicate a broad and generalist diet, which is consistent with previous observations on the basis of stomach content analyses of *P. gibbesi* captured in Argelia and Italy (Puccio et al. 2006; Bada et al. 2022). *Percnon gibbesi* has gastric mills that allow them to consume both soft and hard algae (Puccio et al. 2006) and the leaves, fruits and rhizomes of seagrass *Posidonia oceanica* (Bada et al. 2022, Puentes and Anton 2025), which might explain their versatile macrophyte diet. This is also observed in other species of grapsid crabs such as *Pachygrapsus marmoratus* , which feeds on a variety of macroalgae (Cannicci et al. 2002), as well as other majid crab species, such as *Pisa tetraodon* and *Acanthonyx lunulatus* (Cruz‐Rivera 2001). A terrestrial and freshwater meta‐analysis revealed that generalist exotic herbivores exert significant impacts on native plants but not on exotic plants (Parker et al. 2006); therefore, *P. gibbesi* could cause considerable harm to native macroalgae in the Mediterranean.

Despite their generalist diet, our results suggest that *P. gibbesi* presents a strong feeding preference among the six macroalgal species, consuming more *H. virgatum* (average 63% WW day^−1^), *C. cylindracea* (average 57% WW day^−1^), and *H. scoparia* (average 41% WW day^−1^) than *P. pavonica* (average 13% WW day^−1^), *U. compressa* (average 12% WW day^−1^), or *H. incrassata* (average 7% WW day^−1^). Although some of these species were previously documented in the diet of *P. gibbesi* (such as *C. cylindracea* , *H. scoparia* , *P. pavonica*, and *U. compressa* ), *H. virgatum* —the most consumed algae in this study—and *H. incrassata—*an exotic species from the Caribbean—have not yet been documented as food items for this species of crab.

Feeding choices can be influenced by the nutritional characteristics and quality of the macrophyte tissues (Kennish and Williams 1997). However, in our study, no relationship was found between macroalgae nutritional content (organic %N, %P, and %C and C/N and C/P ratios) and the amount of macroalgae biomass consumed. Similarly, the herbivorous crab *Grapsus albolineatus* does not show feeding preferences on macroalgal species on the basis of the overall nutritional composition (% organic N and C) of eight species of macroalgae (Kennish and Williams 1997). High inorganic carbon content in the fronds of macrophytes can act as a deterrent for some species of herbivores (Jensen 2017). However, this was not the case for *P. gibbesi* , which displayed comparable consumption rates between *H. virgatum* (the macroalgae with the highest carbonate content) and *C. cylindracea* (a fleshy noncarbonate macroalgae), excluding calcium carbonate in the blade tissue as an important driver of the feeding preference of this exotic crab.

Another additional determinant influencing herbivore feeding preferences is the origin of macroalgae, with native species often eliciting preferences over exotic ones (Parker and Hay 2005; Cacabelos et al. 2010). Nevertheless, our observations revealed a lack of correlation between *P. gibbesi* feeding preferences and macroalgae origin, as the consumption rates of native (0.643 ± 0.611 g WW crab^−1^ day^−1^; mean ± SD) and invasive macroalgae (0.638 ± 0.661 g WW crab^−1^ day^−1^; mean ± SD) were similar. The elevated consumption rate of invasive *C. cylindracea* stands in stark contrast to the minor consumption rate of invasive *H. incrassata* . Parker et al. (2006) stated that the evolutionary logic for an exotic herbivore suppressing an exotic primary producer makes sense only if the two exotic species are from different home ranges and thus lack recent coevolutionary history. This is the case for *C. cylindracea* , whose native range is delimited to a narrow area in western Australia and does not overlap with the geographic distribution of *P. gibbesi* in the Atlantic and Pacific. In contrast, *H. incrassata* is a sympatric species with the exotic crabs in the western Atlantic and might be adapted to repel this species of herbivore by, for example, presenting a very thick and leathery tallus that could be difficult to ingest.

The macroalgae *C. cylindracea* is considered one of the most impactful invasive species in the Mediterranean Sea (Anton et al. 2019; Montefalcone et al. 2015; Piazzi et al. 2016) and is ubiquitous throughout the basin. It was suggested that the fast Mediterranean expansion of *P. gibbesi* might be facilitated by the extensive presence of *C. cylindracea* (Marić et al. 2016). This is consistent with the invasion meltdown hypothesis (IMH) (Simberloff and Von Holle 1999), which states that positive feedback among invasive species can facilitate each other's establishment by, for example, one serving as a food source for another, leading to invasional “meltdown” scenarios. On the contrary, *P. gibbesi* was introduced to the Mediterranean recently (~25 years ago; Relini et al. 2000; Garcia Ll 2000; Müller 2001) and the crab may still maintain feeding preferences originating from its native distribution, such as favoring the consumption of *H. scoparia* or *H. virgatum* , which are distributed from the Gulf of Guinea to Madeira (Price et al. 1978; John et al. 2004), akin to the distribution of *P. gibbesi* (Bada et al. 2022).

The feeding preferences of *P. gibbesi* for the three species of macroalgae ( *H. virgatum* , *C. cylindracea* and *H. scoparia* ) found in our study may have been influenced by the morphology of the crab and its habitat preferences. *Percnon gibbesi* are relatively flat and short crabs (barely 0.5 cm tall) that might prefer to eat macroalgae attached to the substrate, forming a moss‐like cover, such as *H. virgatum* , *C. cylindracea* , and *H. scoparia* . Macroalgae like *P. pavonica*, *U. compressa* , and *H. incrassata* , which are attached to the substrate by a small‐area holdfast, may be less accessible for *P. gibbesi* and therefore less preferred. In the case of *P. pavonica* and *U. compressa* , the fleshy blades are often in motion on shallowly exposed rocky shores, which could make their accessibility and ingestion difficult for relatively short *P. gibbesi* . Finally, the three species of preferred macroalgae are frequently found in or near crevices of the rocks and boulders, where *P. gibbesi* are usually found during the day (Authors, personal observation), possibly preferentially feeding on them owing to habitat proximity.

Because of its high per capita and per area grazing rates and generalist diet, *P. gibbesi* could be considered a threat to native Mediterranean species. In fact, two other similar‐sized invasive marine decapods (the Asian shore crab *Hemigrapsus sanguineus* and the European green crab *Carcinus maenas* ) lead first and third in the top ranking of the worst exotic species on the basis of their quantitative ecological impacts (Anton et al. 2019). *Percnon gibbesi* shares ecological niches with many important grazers in the shallow water of the Mediterranean Sea, including crab *Pachygrapsus marmoratus* and sea urchins *P. lividus* and *A. lixula* (Puccio et al. 2006; Sciberras and Schembri 2008). Nonharmful or competitive interactions between the native grapsid *P. marmoratus* and *P. gibbesi* were reported (Sciberras and Schembri 2008). However, *P. lividus* and *A. lixula* were proposed as potential competitors given their similar feeding habitats and their shared ecological niches with *P. gibbesi* (Puccio et al. 2006). The sea urchins *P. lividus* and *A. lixula* are commonly found at shallow sublittoral rocky coastal depths and are recognized as the most common benthic grazers in the Mediterranean Sea (Boudouresque and Verlaque 2013; Privitera et al. 2008). Despite their overlapping habitats, there are slight differences in their feeding niches (Boudouresque and Verlaque 2013; Bonaviri et al. 2011; Bulleri et al. 1999). Multiple studies have analyzed the gut contents of *P. lividus* , demonstrating preferences for coarsely branched erect algae (*P. pavonica*, *Dictyota* spp.), articulated coralline algae ( *Jania rubens* and *H. virgatum* ), and seagrasses such as *P. oceanica* (Boudouresque and Verlaque 2013; Privitera et al. 2008; Chiantore et al. 2008). On the other hand, *A. lixula* prefers encrusting coralline algae (Boudouresque and Verlaque 2013; Bonaviri et al. 2011; Bulleri et al. 1999) and to a lesser extent *Ceramium* spp. and *Polysiphonia* spp. (Privitera et al. 2008). The feeding preferences of *P. gibbesi* overlap mostly with those of the urchin *P. lividus* , as evidenced by the consumption of *H. virgatum* and *P. pavonica* in our experiment and the presence of *Dictyota* spp. and *Jania rubens* in *P. gibbesi* guts (Puccio et al. 2006), suggesting a potential competition for resources between the two herbivores. *Polysiphonia* spp. was also reported in the stomachs of *P. gibbesi* in Italy, which may suggest competition between the urchin *A. lixula* and the exotic crab (Puccio et al. 2006). However, contrasting diets among species are only a first step in determining species competition, and further investigations are needed to fully comprehend the ecological ramifications of the introduction of *P. gibbesi* for Mediterranean native herbivores.

Invasions by marine herbivores are rare. A recent meta‐analysis revealed that the effects of only a few invasive herbivores worldwide have been quantified, with little information on benthic grazers (the impact of more than 85% of herbivore species reported in Anton et al. (2019) are filter‐feeders). A known significant case of marine benthic grazers is the rabbitfish ( *Siganus luridus* and *S. rivulatus* ), which were introduced via the Suez Canal. Rabbitfish can dominate the fish biomass in the eastern Mediterranean, with the capacity to reduce benthic biomass and species richness up to 60% and 40%, respectively (Vergés et al. 2014). Similarly, the exotic gastropod *Zeacumantus subcarinatus* can reduce the macroalgae fronds in the rock pools in southern Australia to bare substratum, affecting as well the abundance of native gastropods (Hendrickx et al. 2015). In this study, we report the potential negative impacts of exotic herbivorous decapods in marine benthic habitats. Given the high densities of *P. gibbesi* found in the field, the large both per capita and per area grazing rates quantified experimentally, and the broad generalist diet reported here and elsewhere (Puccio et al. 2006; Bada et al. 2022), the ecological impacts of *P. gibbesi* on shallow rocky shore ecosystems of the Mediterranean Sea are expected to be severe. Understanding the abundance and feeding preferences of this generalist exotic crab provides a deeper understanding of the mechanisms underlying the invasions of marine herbivores.

## Author Contributions

**Maria Pizarro‐Borrull:** data curation (equal), formal analysis (equal), investigation (lead), writing – original draft (lead), writing – review and editing (lead). **Elisabet Font:** investigation (equal), validation (equal), writing – review and editing (equal). **Núria Marbà:** validation (equal), writing – review and editing (equal). **Andrea Anton:** conceptualization (lead), formal analysis (equal), funding acquisition (lead), investigation (equal), methodology (equal), project administration (lead), resources (equal), supervision (lead), validation (equal), writing – original draft (lead), writing – review and editing (lead).

## Conflicts of Interest

The authors declare no conflicts of interest.

## Data Availability

Data associated to this manuscript is openly available in Dryad here: https://doi.org/10.5061/dryad.vq83bk43w.
